# SCSCTS: An improved SCS+C topographic correction model with shadow compensation for mountainous regions

**DOI:** 10.1371/journal.pone.0347784

**Published:** 2026-04-30

**Authors:** Xuanying Shen, Yutao He, Linlin Chen, Suihua Liu, Zhongyan Wu, Shanhai Song, Lingling Deng, Xiaoyi Du

**Affiliations:** 1 School of Geography and Environmental Sciences, Guizhou Normal University, Guiyang, China; 2 Key Laboratory of Remote Sensing Applications in Mountain Resources and Environment, Guiyang, China; 3 Guizhou Provincial Ecological and Agricultural Meteorological Center, China; Indian Institute of Technology Jammu, INDIA

## Abstract

Topographic effects substantially distort surface reflectance and vegetation indices in mountainous regions, leading to systematic biases in ecological and biophysical parameter retrievals. This study proposes an illumination-stratified topographic correction method (SCSCTS), which extends the conventional Sun-Canopy-Sensor plus C (SCS + C) model by introducing an illumination-based zonal radiative compensation scheme and explicitly accounting for terrain occlusion and adjacent terrain reflection. Using Landsat 8 surface reflectance data from a karst mountainous region in Guizhou, China, the performance of SCSCTS was evaluated and compared with SCS + C and the path length correction (PLC) model under spring and winter conditions. Quantitative results show that, relative to SCS + C, SCSCTS reduced the dependence of near-infrared reflectance on topographic illumination by up to 99.4% under low solar elevation conditions and by 53.8%−67.2% in spring. The proportion of anomalous reflectance pixels decreased by 72.0% in spring and 93.9% in winter, while shaded-sunlit reflectance differences were reduced by 39.8% and 80.8%, respectively. Land-cover-weighted radiometric deviations decreased by 10.3% under high and 24.9% under low solar elevation conditions. At the vegetation index level, SCSCTS reduced terrain-related correlations of EVI, NIRv, and RDVI by 78.6% in spring and 86.1% in winter compared with SCS + C, with regression slopes approaching zero. Compared with PLC, SCSCTS exhibited lower seasonal sensitivity and more stable correction performance under strong shadow conditions. The illumination-stratified correction framework improves radiometric consistency for both reflectance and vegetation indices, suppresses overcorrection effects, and enhances the robustness of remote sensing parameter retrievals in complex mountainous environments. This approach provides a physically based and operationally feasible solution for topographic correction in mountainous remote sensing applications.

## 1. Introduction

Vegetation indices (VIs) are important biophysical indicators widely used in remote sensing to estimate vegetation cover, leaf area index (LAI), biomass, and absorbed photosynthetically active radiation [1–5]. In addition, VIs can reduce the influences of confounding factors such as soil background and atmospheric effects [6–8]. However, the accuracy of these indices fundamentally depends on reliable surface reflectance estimates. In mountainous regions with complex terrain, variations in slope, aspect, and solar incidence angle cause uneven surface illumination, which substantially modifies the surface reflectance and introduces biases into VIs retrieval [9,10]. Previous studies have reported that the average error in remotely sensed leaf area index estimates in regions with slopes reaching 60° could be as high as 51% [11–13]. Given that mountainous areas account for approximately 24% of global land surfaces [14,15], reducing topographic effects in remote sensing imagery is essential for improving the accuracy of reflectance and vegetation indices is crucial for enhancing ecological monitoring in complex terrain.

Numerous topographic correction methods have been developed to mitigate terrain-induced effects in remote sensing imagery. These methods are generally classified as empirical–statistical, semi-empirical, or physical models [10,16]. Empirical–statistical models are typically established using the regression relationships between surface reflectance and topographic variables (e.g., slope, aspect, and solar incidence angle) without explicitly considering radiative transfer mechanisms [16]. Representative approaches include the band ratio method [17], statistical-empirical (SE) model [18], and variable empirical coefficient algorithm (VECA) model [19]. Although these models are computationally efficient and easy to implement, they are often region-specific, lack physical justification, and are prone to overcorrection, which restricts their applicability in areas with highly variable topography and heterogeneous land cover [16]. In contrast, physical models simulate the interactions between solar radiation and surface elements using radiative transfer theory to account for the topographic effects on reflectance. Notable examples include the Minnaert [20], Minnaert + sun-canopy sensor (SCS) [21], and BRDF-based atmospheric and topographic correction (BRATC) [22] models, which incorporate direct, diffuse, and terrain-reflected radiation. Although these models are theoretically robust and can be broadly generalized, they require highly precise input parameters and are computationally demanding [16], which has limited their widespread use in large-scale or data-limited applications [23]. Semi-empirical models provide a compromise by retaining physical plausibility while simplifying radiative transfer processes; thus, they are the most commonly used class of methods in practical applications [18]. Semi-empirical models typically integrate illumination-related topographic factors (e.g., slope, aspect, and solar incidence angle) with surface reflectance using simplified correction functions [14]. Well-known examples include the cosine-correction [16], C-correction [24], SCS [25] and SCS+C [26] models. Among these, the SCS+C model is known for its simplicity and robustness [27] and is one of the most extensively applied topographic correction methods in mountainous remote sensing studies [15,18,28,29].

Topographic shadowing can substantially alter surface reflectance, causing systematic differences in vegetation index (VI) values for the same land-cover type under varying terrain and illumination conditions [10,30,31]. To mitigate these effects, previous studies have proposed terrain-adjusted VIs, such as the topography-adjusted vegetation index (TAVI) [32] and shadow-eliminated vegetation index (SEVI) [31]. These indices introduce terrain-related adjustment factors to reduce the influence of self-shadow and cast shadow, improving index stability in shaded areas. However, they require paired sunlit–shaded samples for parameter estimation, increasing practical complexity, and may compress spectral information, resulting in limited robustness across land-cover types and complex terrain [10,29,33]. To balance physical interpretability and shadow-correction capability, recent studies have attempted to integrate physical modeling with spectral information. Yang et al. [1] developed the NTSEC method, which explicitly compensates for the reduction of direct irradiance and significantly reduces NDVI discrepancies between sunlit and shaded slopes. Jiang et al. [29] introduced the integrated topographic correction (ITC) method, which combines SCS+C with SEVI and uses random forest regression to adjust shaded reflectance and VIs based on their relationships with sunlit samples. Although these approaches improve the correction of shaded areas, their performance is constrained by the transferability of training samples and remains uncertain in highly fragmented karst terrain.

Mountainous karst regions, which are characterized by steep slopes, deeply incised valleys, and highly fragmented terrain with average slopes greater than 20°, present major challenges for topographic correction. In addition, studies on complex mountainous environments have emphasized that accurately restoring reflectance and vegetation indices in shadowed areas is crucial for ecological monitoring [34].These challenges are particularly acute in winter, as extensive shadowing is present in remote sensing imagery. Previous studies have shown that the widely used SCS+C method frequently overcorrects shaded areas [16], resulting in texture distortion and biased reflectance or VI estimates [18]. Such artifacts compromise the accuracy of subsequent ecological parameter estimates and spatial analysis. Previous studies have attempted to reduce the overcorrection problem of the SCS+C model, but its underlying causes are still not fully understood. In shadowed areas of complex mountainous karst terrain, overcorrection is mainly caused by the Lambertian surface assumption and the simplified representation of terrain illumination geometry, which can overestimate the radiance differences between sunlit and shaded slopes. Moreover, the use of a single global C parameter ignores spatial heterogeneity in surface and canopy properties, which may further distort reflectance estimates in shaded regions. Therefore, improving the SCS+C to reduce overcorrection and enhance the accuracy of surface reflectance and vegetation indices remains an important research topic.

In this study, we focused on a typical karst mountainous region in Guizhou, China, and used Landsat 8 OLI surface reflectance data to improve topographic correction performance in shadowed environments. We developed an enhanced SCSCTS model aimed at reducing the overcorrection associated with SCS+C while improving the overall accuracy of reflectance and VIs. Three models (SCSCTS, SCS+C, and PLC) were applied to perform reflectance correction, and NDVI, EVI, NIRv, and RDVI were then calculated from the corrected reflectance. Multiple diagnostic metrics, including visual inspection, statistical correlation and spatial performance comparisons were used to evaluate correction quality and to assess the applicability of the proposed SCSCTS model under rugged terrain and complex illumination. The results provide methodological guidance for topographic correction in mountainous regions and contribute to improving vegetation index retrieval and ecological parameter estimation in complex terrain.

## 2. Materials and methods

### 2.1. Study area

**Fig 1:**
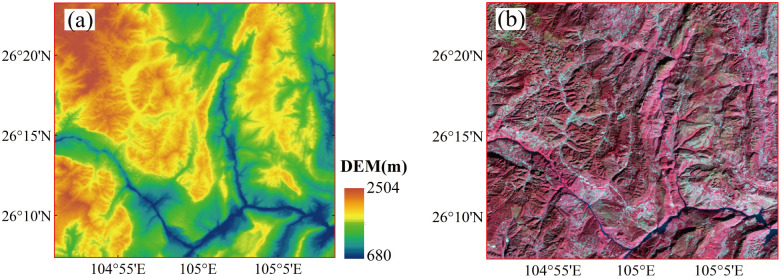
Study area overview. **(a)** Digital Elevation Model (DEM) from Copernicus (ESA); **(b)** False-color composite of Landsat 8 OLI imagery (USGS). Images were processed by the authors to illustrate terrain.

The study area (Fig 1) is located in the mountainous karst region of Liupanshui City, Guizhou Province, southwestern China, covering approximately 30 km × 30 km, within 26°00′–26°30′N and 104°0′–106°00′E. This region is characterized by pronounced topographic variability, with elevations ranging from 680 to 2510 m (average of 1573 m). The terrain includes complex geomorphological features typical of river valley–hill systems, with steep slopes reaching 79.60° (mean slope of 22.75°). The land cover in this region is highly heterogeneous and is dominated by forest ecosystems, croplands, bare land, and water bodies. Substantial terrain-induced illumination differences occur, as the steep slopes and rugged topography frequently obstruct direct solar radiation, thereby resulting in extensive shadowed zones within optical remote sensing imagery. These shaded areas are typically represented by low-reflectance pixels and pose challenges to accurate VIs retrievals. Given these conditions, the selected study area contains a representative landscape for assessing the effectiveness of topographic correction methods, particularly for differentiating their performances between sunlit and shaded terrain, which facilitates robust evaluations of correction accuracy and spatial consistency in complex mountainous environments.

### 2.2. Landsat data

Surface reflectance data from the Landsat 8 OLI were obtained from the United States Geological Survey (USGS) Earth Resources Observation and Science (EROS) Center (https://espa.cr.usgs.gov/). Owing to its balanced spatial, spectral, and temporal resolutions and rigorous quality control, Landsat 8 data are ideal for topographic correction and VIs retrieval in mountainous regions [10]. To compare the correction performances of the methods across seasons, two Landsat 8 OLI scenes from April 16, 2020 (spring), and November 10, 2020 (winter), were selected. Both scenes corresponded to Path 128 and Row 042, with spatial resolutions of 30 m. The spring image had a 63.19° solar elevation angle and 3.41% cloud cover, whereas the winter image had a 43.06° solar elevation angle and only 0.06% cloud cover. We used Level-1 (L1) and Level-2 (L2) Landsat 8 OLI reflectance products in this study. The L2 product, which incorporates atmospheric correction, was used for the SCS+C and PLC topographic correction models, as well as for terrain correction of sunlit areas in the proposed SCSCTS approach. The L1 product was used to construct the compensation term for the shaded areas. Specifically, the L1 reflectance data were initially subjected to radiometric calibration and atmospheric correction. Based on the preprocessed data, the diffuse irradiance from atmospheric scattering and the irradiance reflected from the surrounding terrain were estimated using mountain radiation transfer principles. These irradiance components were then combined to derive a physically based compensation factor for the loss of direct solar radiation in shadowed regions.

### 2.3. Digital elevation models

The Copernicus digital elevation model (DEM) GLO-30, obtained from the European Copernicus Earth Observation Programme, was used to represent the topographic variations in the study area. The dataset was derived from TanDEM-X interferometric synthetic aperture radar (InSAR) measurements and has a reported global average absolute vertical accuracy of 2.57 m, with a relative accuracy that is superior to those of both the SRTM and ASTER DEMs [35,36]. The data are available from the European Space Agency (https://dataspace.copernicus.eu/) at a spatial resolution of 30 m, which is consistent with the Landsat 8 OLI surface reflectance data, thereby minimizing errors caused by a mismatch in spatial resolution. After clipping the DEM to the study area, the slope and aspect layers were extracted for subsequent topographic correction analyses.

### 2.4. Land-cover data

The land-cover dataset used in this study was derived from the GLC_FCS30D product developed by the research team led by Prof. Liangyun Liu at the Aerospace Information Research Institute (Chinese Academy of Sciences). The dataset is available via Zenodo (https://zenodo.org/records/8239305) [37] and has a spatial resolution of 30 m. The GLC_FCS30D product divides global land cover into 35 detailed categories and has an overall accuracy that exceeds 80% [37–39]. The land-cover types within the study area and their corresponding area proportions are summarized in Table 1.

**Table 1:** Percentage Area of Each Land Cover Type.

Land cover class	Area coverage (%)
Coniferous Forest	23.48
Broadleaved Forest	39.72
Grassland	20.90
Cropland	14.34
Impervoious surfaces	0.49
Shrubland	0.34
Water Body	0.60
Marsh	0.07
Flooded flat	0.06
Toal	100.000

### 2.5 Vegetation Indices

To assess the abilities of the two topographic correction methods to mitigate terrain effects on VIs in mountainous conditions, four widely used VIs were selected [6,15] (Table 2). The reflectance values in the NIR, red and blue spectral bands are denoted as $\rho_{NIR}$, $\rho_{R}$ and $\rho_{B}$, respectively.

**Table 2:** Selected vegetation indices and formulas.

VIs	Formula	Reference
Normalized Difference Vegetation Index (NDVI)	$NDVI=\frac{\rho_{NIR}-\rho_{R}}{\rho_{NIR}+\rho_{R}}$	[40]
Enhanced Vegetation Index (EVI)	$EVI=2.5\times \frac{\rho_{NIR}-\rho_{R}}{\rho_{NIR}+6\rho_{R}-7.5\rho_{B}+1}$	[41]
Near-Infrared Reflectance of Vegetation (NIRv)	$NIRv=\rho NIR\times \frac{\rho_{NIR}-\rho_{R}}{\rho_{NIR}+\rho_{R}}$	[42]
Renormalized Difference Vegetation Index (RDVI)	$RDVI=\frac{\rho_{NIR}-\rho_{R}}{\sqrt{\rho_{NIR}+\rho_{R}}}$	[43]

### 2.6. Topographic normalization

Previous studies have shown that the PLC and SCS+C models are the most commonly used and widely validated topographic correction methods, with relatively robust correction performances across various terrain conditions [10,15,44]. Therefore, these two methods were selected as benchmarks for the comparative analysis (Table 3). The SCS+C model is an extension of the original SCS model and assumes that the reflected radiation from sunlit canopy surfaces is proportional to the illuminated area owing to the anisotropic vegetation structure and its tendency to grow toward light sources [26]. The SCS+C model introduces an empirical correction factor C, which is determined from the slope and intercept of a linear regression between the surface reflectance and the cosine of the solar incidence angle. This adjustment enhances the model’s ability to mitigate topographic effects by modifying the SCS equation. In contrast, the PLC model assumes a Lambertian surface and incorporates principles from the radiative transfer equation. The PLC model explicitly accounts for the terrain impacts on the radiation path length within the canopy [44]. By incorporating solar and sensor geometries (including solar zenith, view zenith, and relative azimuth angles), the PLC method calculates relative path lengths on horizontal and inclined surfaces, thereby allowing for more accurate radiative adjustments in areas with complex topography.

**Table 3:** Selected Terrain Correction Methods and Formulas.

Method	Formula	Reference
SCS + C	$\rho_{H}=\rho_{t}\frac{cos\alpha cos\theta s+C}{cosi+C}$	[26]
PLC	$\rho_{H}=\rho_{t}\frac{1/cos\theta_{s}+1/cos\theta_{s}\left(1-tan\alpha cos\left(\varphi_{s}-\beta \right)tan\theta_{s}\right)}{1/cos\theta_{v}+1/cos\theta_{v}\left(1-tan\alpha cos\left(\varphi_{v}-\beta \right)tan\theta_{v}\right)}$	[44]


$$cos\left(i\right)=cos\left(\alpha \right)cos\left(\theta_{s}\right)+sin\left(\alpha \right)sin\left(\theta_{s}\right)cos(\varphi_{s}-\beta )$$ (1)

where $\rho_{H}$ is the corrected reflectance; $\rho_{t}$ is the original reflectance; α is the slope; β is the aspect; C is the empirical parameter; θs and φs represent the solar zenith and solar azimuth angles, respectively; $\theta_{v}$ and $\varphi_{v}$ represent the sensor zenith and sensor azimuth angles, respectively.

### 2.7. SCSCTS correction model

The semi-empirical SCS+C correction method overcorrects in complex mountainous settings characterized by pronounced shadow effects [10,16]. To mitigate this limitation, we enhanced the traditional SCS+C model by incorporating a stratified illumination correction strategy. A radiative transfer model [22] was used to apply radiation energy compensation to reflectance values within shadowed areas based on the differential solar radiation received between illuminated and shaded surfaces. Specifically, the reflectance in sunlit regions was corrected using the conventional SCS+C model, whereas the corrected reflectances of shadowed regions were computed as the sum of the original reflectance and a radiation compensation term (Equation 8). Shadow extraction was performed using the shadow index (SI) method described by Yang et al. [1], which dynamically determines thresholds based on the coastal blue, green, and NIR bands of the Landsat 8 OLI imagery (Equations 3 and 4). This enhanced correction approach is referred to as the SCSCTS model (Equations 2–8).


$$\rho_{d}=\lambda \cdot \rho_{t}\cdot \frac{E_{s}}{E_{w}}$$ (2)

$$E_{w}=E_{k}+E_{a}$$ (3)

$$E_{k}=E_{h}\cdot f\left(\theta_{s},\varphi_{s},\alpha ,\beta \right)$$ (4)

$$E_{a}=\sum_{N}\frac{(L-L_{p})\cdot cosT_{M}cosT_{NdSN}\cdot e^{\tau }/\;cos\theta_{v}}{\overset{\text{2}}{\underset{MN}{r}}}$$ (5)

$$SI=\frac{\rho_{Coastal}-\rho_{Green}}{\left(\rho_{Coastal}+\rho_{NIR})+(\rho_{Green}+\rho_{NIR}\right)}$$ (6)

$$\left\{ \begin{array}{c} @l@\lambda =0\;\;SI<c\\ \lambda =\frac{SI-c}{SI_{max}-c}\;\;SI\geq c \end{array}\right.$$ (7)

$$SI=\frac{\rho_{Coastal}-\rho_{Green}}{\left(\rho_{Coastal}+\rho_{NIR})+(\rho_{Green}+\rho_{NIR}\right)}$$ (8)

where $\rho_{d}\;$ represents the direct solar radiation compensation term, and λ is the coefficient of variation determined from SI. When λ=0 the area is sunlit, whereas λ≠0 denotes a shadowed area. $\rho_{t}$ is the original reflectance and $\rho_{Coastal}$, $\rho_{Green}$, and $\rho_{NIR}$ correspond to the reflectances in the coastal blue, green, and NIR bands, respectively. Es denotes the direct solar irradiance on a horizontal surface and Ew is the irradiance in shadowed areas, which is determined from the sky diffuse Ek and terrain-reflected Ea radiation values. Specifically,$E_{k}$ is equivalent to the horizontal sky diffuse irradiance Eh multiplied by a function (f(θs,φs,α,β)) that is dependent on solar zenith angle θs,solar azimuth angle φs, slope α, and aspect β. L is the radiance measured by the sensor, Lp is the path radiance, τ denotes atmospheric optical thickness, and θv is the sensor zenith angle, dSN represents the pixel area. TM and TN denote the angles between the surface normal and the sensor and between the surface normal and vector MN, respectively, where $r_{MN}$ is the distance between points M and N. $\rho_{Tsun}$ and $\rho_{Tshw}$ are the corrected reflectances for sunlit and shadowed areas, respectively, and $\rho_{SCS+C}$ denotes the reflectance corrected using the SCS + C method.

### 2.8. Model evaluation

Vegetation indices (NDVI, EVI, NIRv, and RDVI) were used only for visual inspection and correlation analysis. The percentage of outliers, spectral consistency between sunlit and shaded slopes, and land-cover radiometry assessments were all performed using surface reflectance values.

(1) Visual analysis

To assess the effectiveness of the different topographic correction methods, visual interpretation was performed by comparing the uncorrected and corrected images. This evaluation was based on the following criteria: (a) the extent to which terrain-induced shadow effects were removed, (b) preservation of spatial texture and fine image details, (c) presence or absence of overcorrection artifacts, and (d) degree to which inter-regional differences were reduced. The visual assessments were used to obtain initial qualitative judgments of the performance and practical applicability of each correction method.

(2) Correlation analysis

The correlation between surface reflectance or vegetation index (VI) values and the cosine of the local solar incidence angle (cos(i)) is widely used to assess the effectiveness of topographic correction [45]. In principle, an effective correction should substantially weaken the dependence of reflectance or VI values on cos(i). Therefore, a linear regression analysis was conducted between cos(i) and the target variable, and both the regression slope and the coefficient of determination (R²) were used as evaluation indicators. Ideally, after correction, the regression slope and R² should approach zero, indicating that terrain-induced radiometric variations have been effectively mitigated. In this study, the correlation analysis was performed for both pre-correction and post-correction conditions using surface reflectance and four vegetation indices, including NDVI, EVI, NIRv, and RDVI. Each variable was analyzed separately.


$$R^{\text{2}}=1-\frac{\overset{N}{\underset{K=1}{\sum }}(X_{k}-{\mathrm{\backslash stackrel}\wedge X}_{k})}{\overset{N}{\underset{K=1}{\sum }}(X_{k}-\overset{―}{X})}$$ (9)

where $X_{k}$ represents the surface reflectance or vegetation index value of pixel k, ${\mathrm{\backslash stackrel}\wedge X}_{k}$ is the value predicted by the linear regression model, $\overset{―}{X}$ is the mean value of $X_{k}$, and N denotes the number of valid pixels.

(3) Percentage of outliers

During topographic correction, some pixels may produce reflectance values outside the valid range of the original image, meaning values higher than the original maximum or lower than the original minimum. These pixels were classified as outliers, and their proportion relative to the total number of pixels was defined as the outlier ratio. A lower outlier ratio indicates better correction performance because it reflects stronger spatial continuity and stability, as well as improved preservation of the physical and spectral properties of surface features [10].


$$OR=\frac{N_{out}}{N_{valid}}$$ (10)

where OR represents the percentage of outliers (%), $N_{out}$ represents the number of outlier pixels whose corrected reflectance values fall outside the valid reflectance range of the original image (i.e., lower than the minimum or higher than the maximum), and $N_{valid}$ represents the total number of valid pixels.

(4) Spectral consistency between sunlit and shaded slopes in rugged terrain

In complex mountainous terrain, substantial differences in incident solar radiation between sunlit and shaded slopes often produce biases in surface radiance or reflectance. The effectiveness of topographic correction and the presence of potential overcorrection were evaluated by comparing the reflectance differences for the same land-cover type between sunlit and shaded slopes [18]. Following established criteria [46], slopes were classified as sunlit when the relative angle between the solar azimuth and slope aspect was less than 45° and shaded when this angle was between 135° and 180°. In addition, areas with slopes steeper than 5° were defined as rugged terrain [6]. A total of 10000 coniferous forest pixels were randomly selected, with 5000 pixels from sunlit slopes and 5000 pixels from shaded slopes. The reflectance differences in the blue, red, and NIR bands before and after correction were computed, and the absolute reflectance differences between sunlit and shaded slopes were used as an indicator of correction performance. This calculation is expressed as follows:


$$D=\left|\rho_{sunlit}-\rho_{shady}\right|$$ (11)

where D denotes the absolute reflectance deviation, $\rho_{sunlit}$ and $\rho_{shady}$ represent the surface reflectance values on sunlit and shaded slopes, respectively.

(5) Land-cover radiometry

The study area was classified into nine land-cover types (Table 1) using the GLC_FCS30D dataset. For each land-cover type, the median reflectance values before and after correction were extracted and the relative differences in median reflectance (RDMR) were calculated (Equation 12). Area-weighted averaging across all land-cover types was then applied to obtain the overall RDMR for each correction method [46]. Ideally, smaller deviations from zero in the area-weighted RDMR indicate that the corrected reflectance is more consistent with true surface characteristics and that the correction performance is better.


$$RDMR_{i}=\frac{\left(\rho_{c,i}-\rho_{u,i}\right)}{\rho_{u,i}}$$ (12)

where $\rho_{u,i}$ and $\rho_{c,i}$ represent the median surface reflectance values of land-cover type i before and after calibration, respectively.

## 3. Results

### 3.1. Reflectance evaluation

#### 3.1.1. Visual analysis.

**Fig 2:**
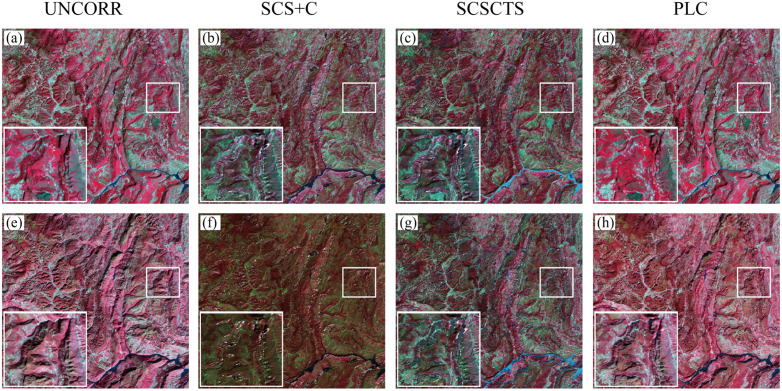
False‐color composite images. Near-infrared (NIR), Red and Green composites for spring (a–d) and winter (e–h) before and after topographic correction.

Fig 2 presents reflectance comparisons for spring and winter under different topographic correction methods, with a magnified view of the area on the right. Overall, terrain effects are most pronounced in winter due to the lower solar elevation angle: illuminated slopes appear excessively bright, shaded slopes appear overly dark, and spatial textures exhibit discontinuities. In contrast, spring imagery is less affected by terrain-induced radiometric variations. After correction, all three methods reduce brightness inconsistencies caused by terrain relief and enhance the spatial uniformity of reflectance to some extent. However, their correction performance differs notably. The SCS+C method shows clear brightness compression in winter, resulting in an overall darkened appearance and local artifacts that disrupt spatial texture continuity, indicating substantial overcorrection under low-incidence-angle conditions. In comparison, PLC and the improved SCSCTS model better preserve the tonal and structural characteristics of the original imagery, and PLC performs best in maintaining spatial texture.

#### 3.1.2. Correlation analysis.

**Fig 3:**
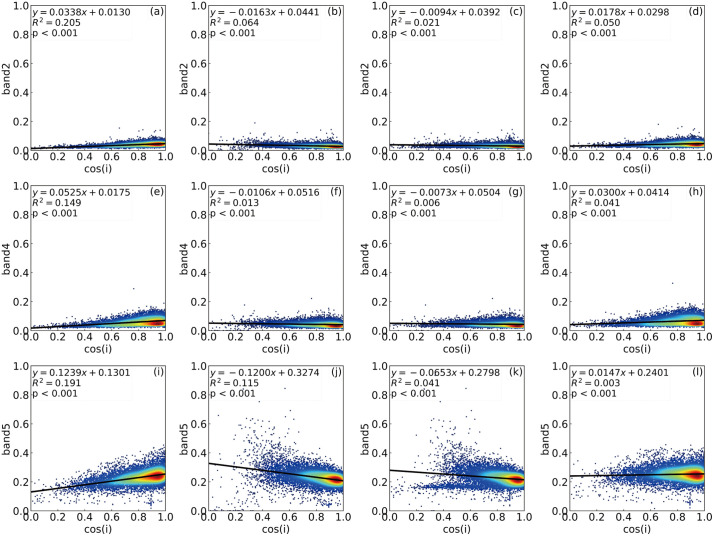
Relationships between reflectance and the cosine of the solar incidence angle in spring. Blue, Red and NIR reflectance before and after topographic correction using the SCS + C, SCSCTS, and PLC models on April 16, 2020. R^2^ denotes the coefficient of determination between reflectance and cos i, where lower values indicate weaker topographic dependence.

**Fig 4:**
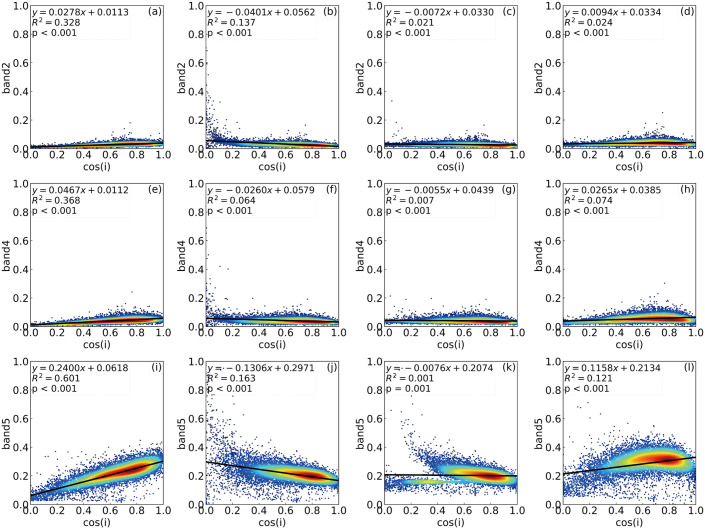
Relationships between reflectance and the cosine of the solar incidence angle in winter. Red and NIR reflectance before and after topographic correction using the SCS + C, SCSCTS, and PLC models on November 10, 2020. R^2^ denotes the coefficient of determination between reflectance and cos i, where lower values indicate weaker topographic dependence.

Figs 3 and 4 show the relationships between surface reflectance in the blue, red, and near-infrared (NIR) bands and the cosine of the local solar incidence angle (cos(i)) before and after topographic correction in spring and winter (The raw data for the reflectance–cos(i) correlation analyses shown in Figs 3 and 4 are provided in S1 File and S2 File, respectively). Overall, terrain effects were most pronounced in winter due to the low solar elevation angle. Strong correlations between reflectance and cos(i) were observed, with R² values of 0.328, 0.368, and 0.601 for the blue, red, and NIR bands, respectively. Among the three bands, the NIR band exhibited the strongest terrain dependence, which can be attributed to its relatively low sensitivity to diffuse skylight and its higher responsiveness to illumination geometry changes [6,44,47]. In contrast, terrain effects were weaker in spring, and R² values were substantially lower across all bands, although the blue band still showed moderate sensitivity (R² = 0.205).

For the spring imagery, all topographic correction methods effectively reduced the correlations between reflectance and cos(i). Compared with the original data, the SCSCTS method reduced the R² values of the blue, red, and NIR bands to 0.021, 0.006, and 0.041, representing reductions of approximately 67.2%, 53.8%, and 64.3% relative to the SCS+C method. The SCS+C method also weakened the terrain dependence in spring (R² = 0.064 for blue and 0.013 for red), but a noticeable residual terrain effect remained in the NIR band (R² = 0.115). The PLC method yielded higher R² values than SCSCTS across all three bands.

In the winter imagery, the differences among correction methods became more evident. The SCSCTS method achieved the best performance, reducing the R² values of the blue, red, and NIR bands to 0.021, 0.007, and 0.001, respectively. These values correspond to reductions of 84.7%, 89.1%, and 99.4% compared with SCS+C, and the regression slopes were close to zero, indicating that the dependence of reflectance on illumination conditions was nearly eliminated. In contrast, SCS+C showed limited correction performance in winter, particularly in the NIR band, where the R² value remained as high as 0.163 and the regression slope became negative, suggesting overcorrection in shaded areas. The PLC method exhibited intermediate performance, with an R² of 0.121 in the NIR band, indicating a residual terrain dependence.

#### 3.1.3. Percentage of outliers.

**Fig 5:**
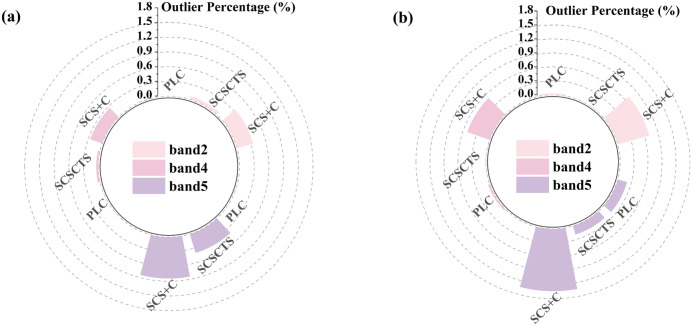
Percentages of outlier reflectance pixels under different topographic correction methods. **(a)** April 16, 2020; **(b)** November 10, 2020.

Fig 5 (The raw data for the reflectance outlier percentages shown in Fig 5 are provided in S3 File) shows the percentages of outliers in reflectance for the blue, red, and NIR bands in April and November for the three topographic correction methods. Overall, the proportions of outliers produced by PLC and SCSCTS remained below 1% in both spring and winter, with PLC exhibiting the most stable performance. Under high solar elevation conditions in spring, PLC generated almost no outliers across all three spectral bands. Even under low solar elevation in winter, the proportions of outliers for the blue and red bands remained close to zero, with only a moderate increase in the NIR band. In contrast, the SCS + C model produced substantially higher percentages of outliers, particularly under low solar elevation conditions, and the proportion of anomalous pixels increased with terrain complexity. For the NIR band, the outlier proportions reached 0.844% in spring and 1.337% in winter, indicating pronounced overcorrection in shaded areas and abnormal reflectance fluctuations.

The proposed SCSCTS model markedly reduced the occurrence of outliers relative to SCS+C. Quantitatively, in spring, SCSCTS reduced the outlier proportions in the blue, red, and NIR bands by 80.6%, 83.0%, and 52.1%, respectively, with an average reduction of approximately 72.0%. In winter, the improvements were even more pronounced, with reductions of 98.0%, 98.3%, and 85.5%, corresponding to an average reduction of 93.9%. These results demonstrate that the illumination-based zonal compensation strategy effectively suppresses the overcorrection effects of SCS+C under strong shadow conditions and substantially improves radiometric stability.

Regarding seasonal variation, the blue band consistently exhibited the lowest proportion of outliers in both seasons, indicating a relatively weak sensitivity to terrain effects. Conversely, the NIR band produced the highest proportion of outliers, consistent with the correlation analysis, reflecting its strong susceptibility to topographic influences. Overall, the proportion of outliers was significantly higher in winter than in spring, further confirming that terrain effects become more pronounced as solar elevation decreases.

#### 3.1.4. Reflectance differences between shaded and sunlit slopes before and after correction.

**Fig 6:**
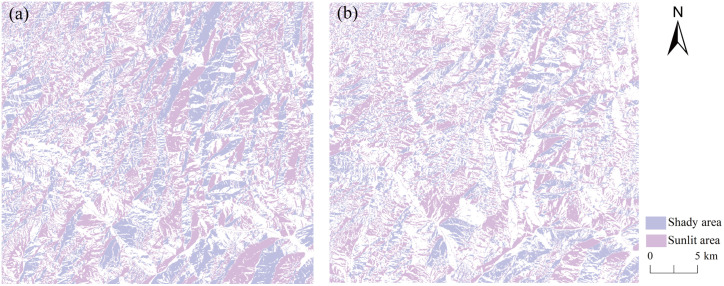
Distribution of shaded and sunlit slopes in rugged terrain. **(a)** April 16, 2020; **(b)** November 10, 2020.

To evaluate the performance of different terrain correction methods under varying slope aspects, reflectance differences between rugged shaded and illuminated slopes were compared for the same land cover type. This metric provides a direct assessment of each method’s ability to reduce aspect-induced radiometric discrepancies. Fig 6 shows the spatial distribution of rugged shaded and illuminated slopes in spring and winter, extracted using QGIS. The satellite overpass occurred on 16 April 2020 at 03:27 UTC (11:27 local time; Fig 6a) and on 10 November 2020 at 03:28 UTC (11:28 local time; Fig 6b).

**Fig 7:**
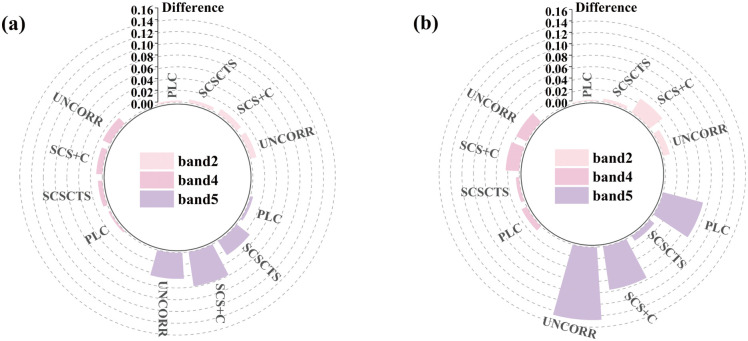
Absolute differences in reflectance between sunlit and shaded slopes before and after topographic correction. Corrections were performed using the SCS + C, SCSCTS, and PLC models. **(a)** April 16, 2020; **(b)** November 10, 2020.

Fig 7 presents the absolute reflectance deviations between shaded and illuminated coniferous forest pixels before and after correction for the blue, red, and near-infrared (NIR) bands (The raw data for the absolute reflectance deviations shown in Fig 7 are provided in S4 File). The NIR band was most strongly affected by terrain effects, showing substantially larger differences between shaded and illuminated slopes, whereas the blue and red bands exhibited relatively smaller deviations. After terrain correction, both the PLC and SCSCTS methods substantially reduced the reflectance differences between slope aspects and showed improved radiometric consistency. In contrast, the SCS+C method exhibited overcorrection in most cases, with larger shaded–illuminated differences after correction than before correction. Compared with SCS+C, the SCSCTS model significantly reduced absolute deviations across seasons and spectral bands. Quantitatively, in the spring scene, SCSCTS reduced the absolute deviations by approximately 40.1%, 27.6%, and 51.6% in the blue, red, and NIR bands, respectively, with an average reduction of 39.8%. In the winter scene, the improvement was more pronounced, with reductions of approximately 80.3%, 75.6%, and 86.6%, and an average reduction of 80.8%. These results indicate that the proposed method substantially improves aspect-related radiometric consistency under strong terrain shading conditions and outperforms the conventional SCS+C model. The PLC model performed relatively well under high solar elevation conditions in spring, but its ability to compensate for illumination differences in rugged terrain weakened under low solar elevation conditions in winter. Large shaded–illuminated differences remained in the NIR band after correction, indicating a strong seasonal dependence. Under low solar elevation angles, PLC tends to underestimate the optical path length for shaded slopes, which limits its radiometric consistency in complex terrain [18].

#### 3.1.5. Stability of land-cover radiometry.

**Fig 8:**
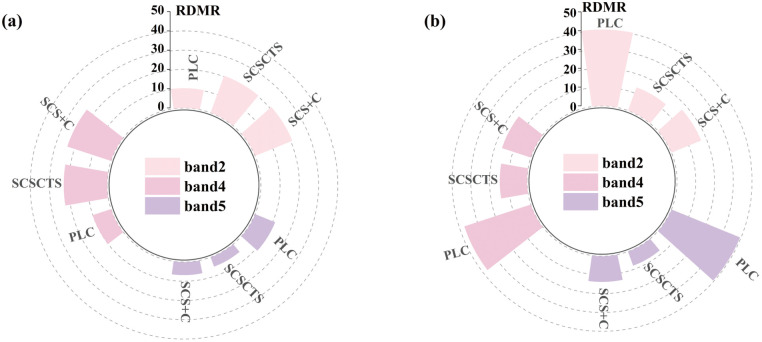
Weighted RDMR values of median reflectance under different topographic correction methods. Results are summarized by land-cover types. **(a)** April 16, 2020; **(b)** November 10, 2020.

Fig 8 shows the relative differences in land-cover–weighted median reflectance (RDMR) for April and November, which were used to evaluate the ability of the three terrain correction methods to reduce radiometric discrepancies among land-cover types. The raw data for the land-cover–weighted RDMR values shown in Fig 8 are provided in S5 File. Overall, differences among the methods were smaller in April, when the solar elevation angle was high and topographic effects were relatively weak, whereas method-dependent differences became more pronounced in November due to stronger terrain effects under low solar elevation conditions. In April, PLC exhibited the lowest RDMR values in the blue and red bands, indicating good radiometric stability in the visible spectrum, while SCS+C and SCSCTS showed comparable performance. However, PLC produced the highest RDMR in the NIR band, suggesting limited correction capability for terrain-sensitive wavelengths. Compared with SCS+C, SCSCTS showed consistent improvements across all three bands, with RDMR reductions of approximately 1.7%, 5.5%, and 23.8% in the blue, red, and NIR bands, respectively, corresponding to an average reduction of about 10.3%. These results indicate that the proposed method enhances radiometric consistency even under high solar elevation conditions. Under low solar elevation conditions in November, the performance differences among the methods increased substantially. SCSCTS achieved the lowest RDMR values in the blue, red, and NIR bands, demonstrating the highest radiometric stability. Quantitatively, SCSCTS reduced RDMR by approximately 23.0%, 10.0%, and 41.6% relative to SCS+C, with an average reduction of 24.9%. This highlights the strong capability of the proposed method to mitigate radiometric discrepancies among land-cover types under complex illumination conditions and its improved adaptability to rugged terrain. In contrast, PLC exhibited RDMR values close to 40% across all three bands, indicating pronounced overcorrection and strong seasonal sensitivity, with a marked decrease in stability during winter. Overall, compared with the conventional SCS+C method, the proposed SCSCTS approach more effectively reduces radiometric differences among land-cover types, improves post-correction radiometric consistency, maintains low RDMR values across seasons, and shows the weakest seasonal dependence, indicating more robust and stable terrain correction performance.

### 3.2. Vegetation index assessment

In addition to the reflectance-based evaluation, four vegetation indices (NDVI, EVI, NIRv, and RDVI) were calculated in spring and winter to further assess the performance of topographic correction at the vegetation index level. Since vegetation indices are derived from multi-band combinations and are often less sensitive to illumination differences due to their ratio-based formulations, their evaluation requires different considerations compared with surface reflectance. Therefore, this study adopted visual inspection and correlation analysis, which are commonly used approaches for assessing the effectiveness of terrain-effect removal in vegetation indices [10]. The analysis was conducted for three correction methods (SCS + C, SCSCTS, and PLC).

#### 3.2.1. Visual analysis.

**Fig 9:**
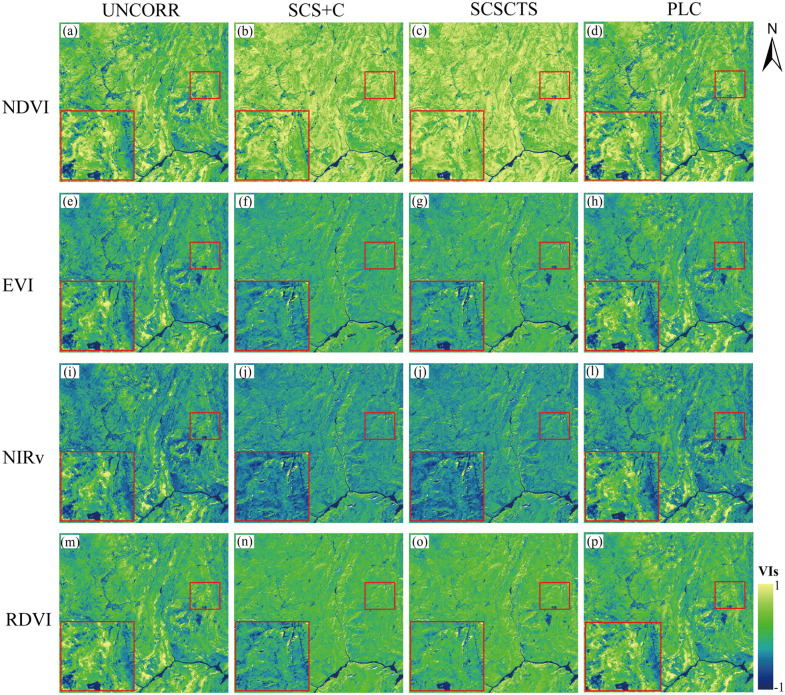
Vegetation indices in spring before and after topographic correction. NDVI, EVI, NIRv, and RDVI derived on April 16, 2020 using the SCS + C, SCSCTS, and PLC models.

**Fig 10:**
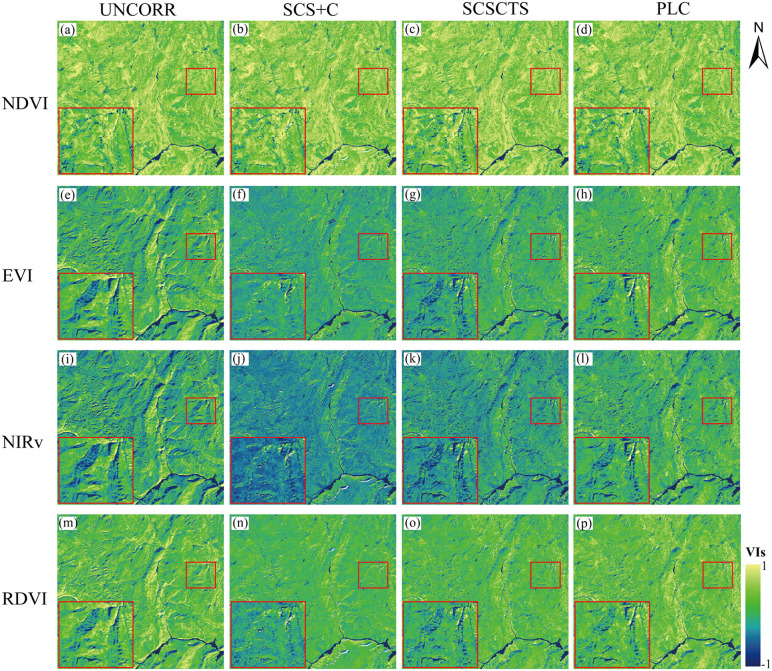
Vegetation indices in winter before and after topographic correction. NDVI, EVI, NIRv, and RDVI derived on November 10, 2020.

Figs 9 and 10 show the NDVI, EVI, NIRv, and RDVI images before and after applying the three terrain correction methods in spring and winter. Overall, the responses of the vegetation indices to terrain conditions were generally consistent with those of reflectance. Terrain effects were relatively weak in spring and became more pronounced in winter. All three terrain correction methods reduced the spatial differences caused by slope and aspect to varying degrees and improved the spatial continuity of the images. In both seasons, NDVI exhibited the smallest changes in texture and brightness after correction, indicating its relatively low sensitivity to terrain effects. For EVI, NIRv, and RDVI, the SCS+C method caused an overall decrease in vegetation index values and produced localized pixel fragmentation and texture loss in winter, which is related to its tendency toward overcorrection in shaded areas [44]. Under low solar elevation angles, such overcorrection suppresses the brightness of both illuminated and shaded areas and weakens spatial texture continuity. In contrast, the SCSCTS model effectively removed terrain effects while maintaining reasonable gradients in sunlit areas, leading to a more natural and spatially consistent distribution in the corrected images. The PLC method also substantially reduced the differences between shaded and sunlit slopes and preserved the spatial texture without producing obvious brightness suppression or fragmentation.

The enlarged subregions in the lower-left corner further illustrate these effects. In winter, the original images showed the strongest contrast between sunlit and shaded areas, particularly in EVI and NIRv. Although the SCS+C method reduced these differences, the vegetation index values of the illuminated areas were noticeably underestimated, resulting in blurred textures. The SCSCTS model not only reduced the slope-related differences more effectively, but also preserved the numerical characteristics of the illuminated areas, producing higher continuity and visual realism after correction. The PLC method exhibited similar performance, reducing terrain effects while maintaining coherent spatial textures.

#### 3.2.2. Correlation analysis.

**Fig 11:**
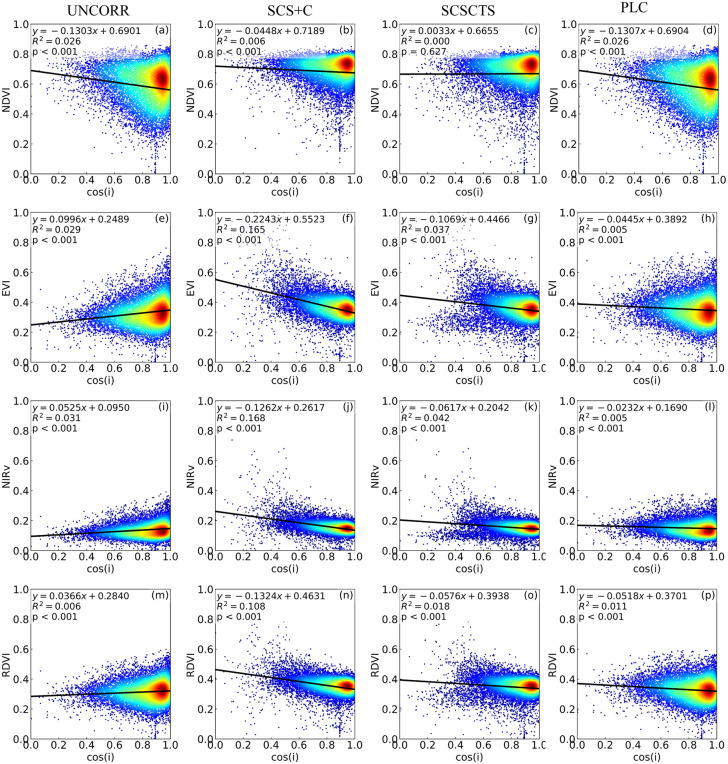
Relationships between vegetation indices and the cosine of the solar incidence angle in spring. NDVI, EVI, NIRv, and RDVI before and after topographic correction on April 16, 2020. R^2^ represents the coefficient of determination between vegetation indices and cos i, with lower values indicating improved topographic correction.

**Fig 12:**
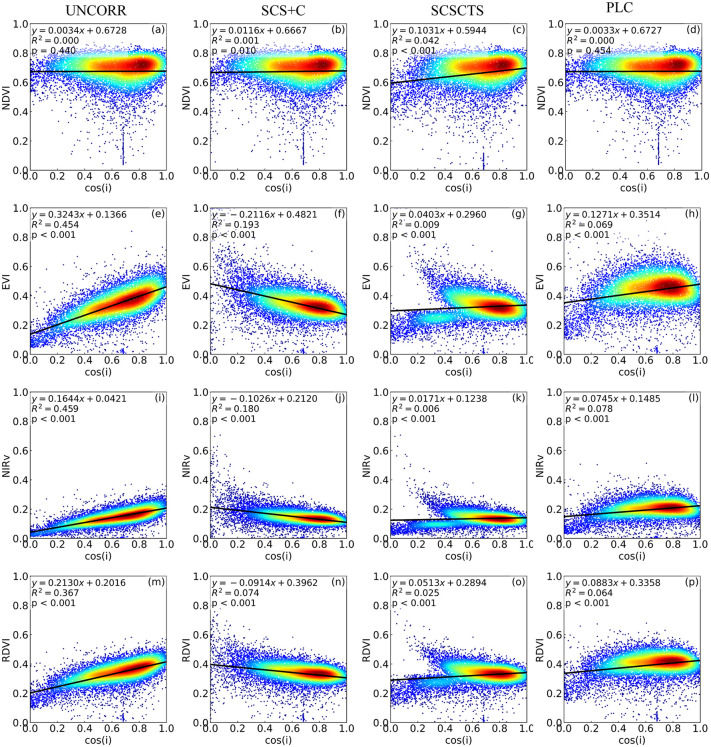
Relationships between vegetation indices and the cosine of the solar incidence angle in winter. NDVI, EVI, NIRv, and RDVI before and after topographic correction on November 10, 2020. R^2^ represents the coefficient of determination between vegetation indices and cos i, with lower values indicating improved topographic correction.

The correlations between the four vegetation indices and the local cos(i) in spring and winter are shown in Figs 11 and 12. Overall, NDVI was the least sensitive to terrain effects, with R² values between NDVI and cos(i) close to zero in both seasons, indicating that terrain-induced illumination differences had a negligible influence on NDVI. The raw data for the vegetation index–cos(i) correlation analyses shown in Figs 11 and 12 are provided in S6 File and S7 File, respectively. This insensitivity is mainly attributable to the strong correlation between red and near-infrared reflectance, which allows the normalized ratio formulation of NDVI to offset most terrain-induced radiometric variations [6,15,18,45]. Notably, in winter, the R² values of NDVI increased after SCS+C and SCSCTS corrections, suggesting that ratio-based vegetation indices already mitigate terrain effects to a large extent prior to correction, and further adjustments may introduce additional disturbances [6]. In contrast, EVI, NIRv, and RDVI were more sensitive to terrain conditions and solar elevation angle. In spring, when the solar elevation angle was high, their correlations with cos(i) were generally weak (R² < 0.1), and regression slopes were close to zero. After correction, the SCS+C method exhibited overcorrection in some areas, whereas SCSCTS and PLC showed comparable overall performance. Quantitatively, compared with SCS+C, SCSCTS further reduced the R² values of EVI, NIRv, and RDVI by an average of 78.6% in spring, indicating that the proposed method can further weaken terrain dependence even under high solar elevation conditions.

In winter, when the solar elevation angle was low, all three vegetation indices showed significant positive correlations with cos(i), indicating that terrain-induced radiometric interference was substantially amplified [15,18]. Among them, NIRv exhibited the strongest correlation (R² = 0.459), followed by EVI (R² = 0.454) and RDVI (R² = 0.367). Although SCS+C reduced the R² values, the regression slopes became negative and vegetation index values were abnormally high in low cos(i) regions, reflecting a typical overcorrection effect. By contrast, the SCSCTS model showed clear advantages in winter, reducing the R² values of NIRv, EVI, and RDVI to 0.0171, 0.0403, and 0.0513, respectively. Compared with SCS+C, SCSCTS achieved an average R² reduction of 86.1% across the three indices, substantially weakening the linear dependence of vegetation indices on terrain illumination conditions. Moreover, regression slopes approached zero, indicating that illumination effects were effectively removed while avoiding local overcorrection. The PLC method also produced stable corrections, with notable decreases in both R² and slope, although its overall improvement was slightly weaker than that of SCSCTS.

Overall, SCSCTS demonstrated a more consistent capability to reduce terrain effects across seasons, significantly improving the robustness and reliability of vegetation index retrievals in complex mountainous regions.

## 4. Discussion

### 4.1. Comparing the SCSCTS with Existing Approaches

In rugged mountainous regions, surface illumination conditions vary substantially with seasonal changes in solar zenith angle [48]. This study proposes an improved SCSCTS approach based on the traditional SCS+C model, which introduces an illumination-based zonal radiative compensation mechanism that explicitly accounts for slope, aspect, solar–sensor geometry, diffuse skylight, and adjacent terrain reflection [49,50].

Quantitative evaluations demonstrated that SCSCTS consistently outperformed conventional SCS+C and PLC methods. For reflectance correction, SCSCTS reduced the dependence of reflectance on cos(i) by up to 99.4% in the NIR band under low solar elevation conditions, and achieved reductions of 67.2-64.3% in spring relative to SCS+C. In addition, SCSCTS reduced the proportion of outliers by 72.0% in spring and 93.9% in winter, indicating a substantial suppression of overcorrection artifacts. Aspect-related radiometric inconsistencies were also markedly improved. Compared with SCS+C, SCSCTS reduced shaded–sunlit reflectance deviations by 39.8% in spring and 80.8% in winter, and decreased land-cover–weighted RDMR by 10.3% and 24.9% under high and low solar elevation conditions, respectively. These results demonstrate that the proposed method enhances radiometric consistency across both slope aspects and land-cover types, particularly under strong terrain shading. At the vegetation index level, SCSCTS further weakened terrain dependence. Relative to SCS+C, the average R² of EVI, NIRv, and RDVI decreased by 78.6% in spring and 86.1% in winter, with regression slopes approaching zero. This indicates that SCSCTS effectively removed illumination-induced biases while avoiding the overcorrection observed in SCS+C. Compared with PLC, which primarily adjusts optical path length and exhibited increased seasonal sensitivity, SCSCTS maintained stable correction performance across illumination conditions. The improved robustness can be attributed to its explicit treatment of shaded-area radiative compensation, which mitigates the systematic overcorrection of SCS+C.

Compared with the NTSEC method proposed by Yang et al. [1], which incorporates terrain occlusion, scattering, and adjacent terrain reflection more explicitly when constructing its shading compensation term. Since NTSEC relies primarily on simplified Vd and Ct parameters, its applicability may be limited in karst environments where topographic fragmentation is high [1,51]. Similarly, the SEVI index can suppress self-shadowing and cast shadows without requiring DEMs or atmospheric parameters, has a relatively simple determination method, and is more effective than most traditional approaches. However, the terrain adjustment factor used to calculate SEVI is determined through a correlation-based balancing approach, and its accuracy depends heavily on the terrain complexity and ratio of shaded to sunlit slopes [31]. The ITC approach integrates SCS+C and SEVI and uses random forest regression to build compensation relationships for shaded areas. It is well suited for low elevation mountainous regions with moderate relief in coastal Fujian (average elevation below 500 meters), but its dependence on large numbers of clear sky samples may limit its transferability and physical interpretability [29].

Previous studies have focused mainly on low elevation regions between 0 and 1000 meters and tend to analyze imagery acquired under favorable illumination, while research on spring (February to April) and winter (January, November, and February) remains relatively limited [10]. In contrast, the study area examined here has an average elevation of 1573 meters, with stronger terrain fragmentation and more pronounced seasonal illumination differences. Under these conditions, the use of pixel level radiometric geometry and adjacent terrain reflected irradiance in this study provides a physically based compensation framework that helps address the lack of terrain correction research for high elevation and strongly shaded environments [22]. It is worth noting that recent work in sustainable environmental assessment [34] emphasizes the need for accurate surface parameters, realistic reflectance retrieval, and consistent model performance across seasons when building ecological monitoring models in complex mountains. Although such studies do not directly address terrain correction, they underscore the importance of improving spectral accuracy in rugged regions and offer a theoretical basis supporting the enhanced shading compensation mechanism developed in this study.

Overall, the proposed SCSCTS model integrates radiative transfer concepts and explicitly considers terrain occlusion and adjacent terrain reflection at the pixel scale. It effectively reduces the overcorrection issue observed in SCS+C within shaded regions and maintains greater stability in complex karst terrain. The method does not require separate sampling of sunlit and shaded slopes, is computationally straightforward, and offers strong practical value and potential for broader application. This study provides a physically grounded and operationally feasible approach for terrain correction of remote sensing data in complex mountainous environments.

### 4.2. Interpretation of topographic correction performance

The texture distortions and higher proportion of outliers produced by the SCS+C model indicate that this method still suffers from overcorrection in rugged terrain and shadowed areas. This finding is consistent with previous studies by Sola et al., suggesting that the limitations of the SCS+C model are broadly applicable. After applying the SCSCTS correction, the relationship between near-infrared reflectance, vegetation indices, and the local solar incidence angle cos(i) in November became more dispersed. This pattern can be attributed to the strong terrain influence on the NIR band and the large solar zenith angles during this period, which result in extensive shadowed areas. Because the SCS+C model applies the same correction strategy to both shaded and unshaded regions, it tends to amplify errors in shadowed areas [18]. In contrast, the improved SCSCTS model effectively mitigates this issue, leading to clearer differences between high and low cos(i) values in the correlation plots. The reduced dispersion observed in April further supports the role of shadow extent in controlling correction performance, indicating the robustness of the proposed approach.

**Fig 13:**
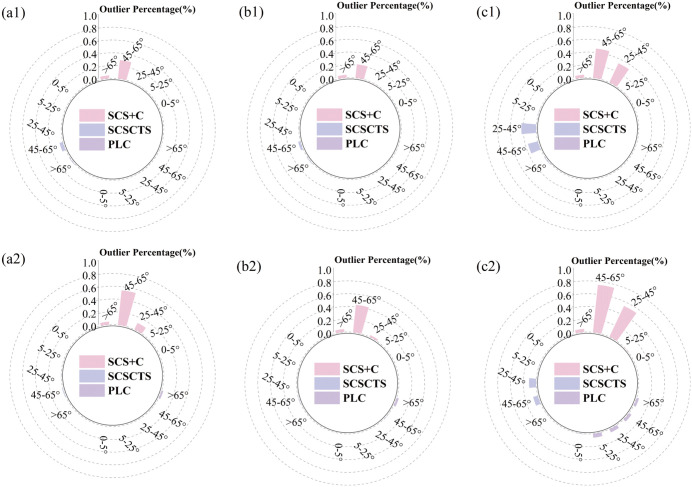
Outlier pixels across different slope classes in April (1) and November (2). Blue **(a)**, Red **(b)**, and NIR (c) reflectance bands are shown.

To interpret the influence of slope on correction performance, the study area was classified into five slope categories (0-5°, 5-25°, 25-45°, 45-65°, and >65°, Fig 13. The corresponding raw data are provided in S8 File). The concentration of anomalous pixels on steep slopes suggests that interactions between self-shadowing and cast shadows in complex terrain may introduce additional uncertainties in topographic correction [18]. Although the PLC method produced fewer anomalous pixels, it exhibited the highest weighted RDMR for land cover in November, likely because it neglects diffuse sky radiation and terrain-reflected radiation, which can introduce errors when estimating path length on steep or shaded slopes [6,18]. This result differs from some previous studies [15,44], which primarily used spring or summer imagery with higher solar elevations and fewer shadowed areas [10]. The comparison between April and November imagery in this study further demonstrates that lower solar elevation angles increase shadow coverage and reduce the effectiveness of the PLC method, consistent with the findings of Ma et al. [18]. These results indicate that the applicability of PLC is limited in shadow-dominated conditions. Among the nine land-cover types, coniferous forest, broadleaf forest, and grassland exhibited the highest RDMR values, which may be related to the Lambertian assumption adopted by all correction methods, ignoring the anisotropic reflectance behavior of vegetation canopies [10,16]. Although the SCSCTS model relies on empirically determined threshold parameters, it consistently improved correction performance across seasons, particularly under low solar elevation conditions, suggesting that the proposed approach is robust and broadly applicable.

It should be noted that SCSCTS inherits the Lambertian surface assumption, which may introduce uncertainties over complex surfaces, particularly in snow-covered or non-vegetated high-reflectance areas where strong anisotropic reflectance and multiple-scattering effects are pronounced [10,52–55]. In addition, the land-cover-weighted RDMR metric does not explicitly differentiate spectral characteristics among land-cover types, which may mask heterogeneous responses to topographic correction across different surface classes [56,57].

## 5. Conclusions

This study proposes an illumination-stratified topographic correction method (SCSCTS), which extends the conventional SCS+C model by introducing an illumination-based zonal radiative compensation scheme and explicitly accounting for terrain occlusion and adjacent terrain reflection. The proposed approach aims to reduce terrain-induced radiometric distortions in surface reflectance over complex mountainous regions.

Quantitative evaluations indicate that SCSCTS outperforms the conventional SCS+C and PLC methods across multiple reflectance and vegetation-index metrics. For surface reflectance, SCSCTS improved the SCS+C method in several aspects: (1) under low solar elevation conditions, the dependence of near-infrared reflectance on the cosine of the solar incidence angle was reduced by up to 99.4%, and by 53.8%-67.2% under spring conditions; (2) the proportion of anomalous reflectance pixels decreased by 72.0% in spring and 93.9% in winter, indicating a substantial suppression of overcorrection artifacts; and (3) shaded–sunlit reflectance differences were reduced by 39.8% in spring and 80.8% in winter, while land-cover–weighted RDMR values decreased by 10.3% under high solar elevation and 24.9% under low solar elevation, demonstrating improved radiometric consistency across slope aspects and land-cover types.

At the vegetation index level, SCSCTS further weakened terrain-related illumination effects. Compared with SCS+C, the average R² values of EVI, NIRv, and RDVI were reduced by 78.6% in spring and 86.1% in winter, with regression slopes approaching zero, indicating that illumination-driven biases were effectively mitigated while avoiding the overcorrection observed in SCS+C. Compared with PLC, SCSCTS exhibited lower seasonal sensitivity and more stable correction performance under strong shadow conditions.

Overall, the illumination-stratified correction strategy substantially improves topographic correction in rugged terrain, particularly under low solar elevation conditions. The SCSCTS method enhances radiometric consistency for both reflectance and vegetation indices, suppresses overcorrection effects, and improves the robustness of remote sensing parameter retrievals in complex mountainous environments. These findings provide a physically based and operationally feasible framework for terrain correction and support more reliable ecological and environmental monitoring applications in mountainous regions.

## Supporting information

S1 File Raw data for the April reflectance–cos(i) correlation analysis shown in Fig 3. (XLSX)

S2 File Raw data for the November reflectance–cos(i) correlation analysis shown in Fig 4. (XLSX)

S3 File Raw data for reflectance outlier percentages shown in Fig 5. (XLSX)

S4 File Raw data for reflectance absolute deviations between sunlit and shaded slopes shown in Fig 7. (XLSX)

S5 File Raw data for land-cover-weighted reflectance RDMR shown in Fig 8. (XLSX)

S6 File Raw data for the April vegetation index–cos(i) correlation analysis shown in Fig 11. (XLSX)

S7 File Raw data for the November vegetation index–cos(i) correlation analysis shown in Fig 12. (XLSX)

S8 File Raw data for reflectance outlier percentages under different slope classes shown in Fig 13. (XLSX)
